# The Role of Point-of-Care Ultrasound in Pilonidal Sinus Disease

**DOI:** 10.24908/pocus.v7i2.15543

**Published:** 2022-11-21

**Authors:** Hadiel Kaiyasah, Lamis Abufool, Labib Al Ozaibi

**Affiliations:** 1 Department of General Surgery, Colorectal Unit, Rashid Hospital Dubai United Arab Emirates

**Keywords:** pilonidal sinus, ultrasound, surgical excision, recurrence, pits

## Abstract

Pilonidal sinus is a common problem encountered in proctology clinics. It has a wide spectrum of clinical picture ranging from a single asymptomatic pit to a more complex disease with multiple sinuses and secondary openings. Hence, the treatment options could range from observation or simple excision to a more radical approach like flap surgeries. Ultrasonographic assessment could help in mapping the extent of the pilonidal sinus. It can also identify whether the sinus is infected or has formed an abscess. With the above-mentioned information provided by the point of care ultrasound, the surgeon can tailor the surgical approach to each individual case and improve the overall outcome. In this article, we are highlighting some examples of cases managed in our proctology unit where ultrasound was done preoperatively and guided the management.

## Introduction

The term “pilonidal” was given by Richard Hodges as a description of the pilus and nidus, or hair and nest 1. The term refers to a condition consisting of one or more openings usually found in the natal cleft overlying the coccyx which communicate with a fibrous track lined by granulation tissue and contains hair within the lumen. The disease is 2.2 times more common in men than in women  2. Presentation can range from asymptomatic openings, acute inflammatory processes presenting as painful abscesses, or chronic sinus with intermittent discharge from the affected area.

The ideal management and surgical treatment of pilonidal sinus disease is still debatable. Depending on the clinical presentation and surgeon’s assessment, the surgical approach can range from simple incision to flap surgeries. 

Ultrasound scanning is an undervalued non-invasive method of examination widely used in medicine. Endoluminal ultrasonography was first introduced in 1989 for the assessment of anal and rectal disorders by Law and Bartram, and has since become essential in coloproctological practice 3.

Ultrasonography is crucial in assessing different anorectal pathologies, mainly abscesses, fistulas, and sphincter defects. In our center, preoperative point of care ultrasound (POCUS) is performed by the same surgeon who will be operating on the patient. This practice, in comparison to departmental ultrasounds done by radiologists, yields better post-operative results and lower rates of recurrence. Here we present examples where bedside POCUS was done for pilonidal sinus disease cases and how it helped in guiding the operative management for optimal patients’ outcome.

## Case 1: Pilonidal abscess

**Figure 1:**
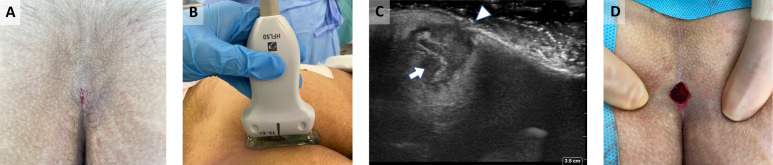
Infected pilonidal cyst. A)Natal cleft with small opening. B)Using high frequency linear probe with a layer of gel to scan the natal cleft area. C)Ultrasonographic sagittal view showing the pilonidal cyst and hair within (arrow) and the single pit (arrow head). No craniocaudal extension. D)Post excision.

A 23-year-old female presented with a history of acute pain at the natal cleft area. POCUS revealed a small pilonidal cyst with no craniocaudal extension. The patient underwent localized excision (Figure 1).

## Case 2: Complex pilonidal sinus

**Figure 2:**
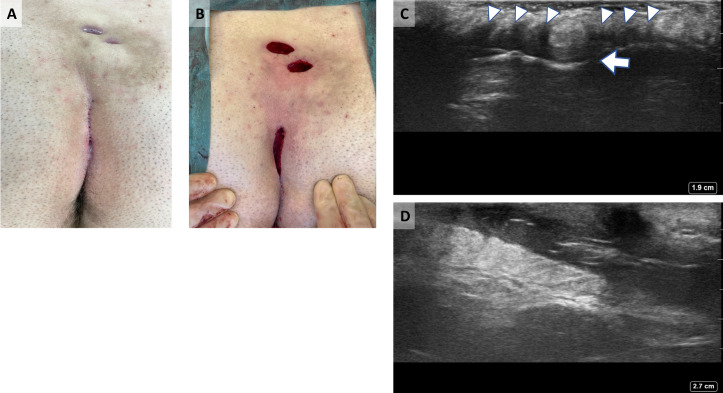
Complex pilonidal Sinus. A) Multiple midline pits and two secondary opening cranially. B)Post modified Lord Millar excision. C) Ultrasonographic view at the midline of natal cleft showing multiple pits (arrowheads) and the pilonidal sinus with hair within (arrow). No craniocaudal extension. D)Ultrasonographic view at the upper part of the natal cleft showing the cranial extension of the pilonidal sinus towards the secondary openings.

A 19-year-old male presented with a long-standing history of discharge from two openings at the upper part of the natal cleft. He had multiple pits at the midline. POCUS showed the sinus tract extending from the midline up to the secondary openings. A modified Lord Millar excision was done (Figure 2).

## Case 3: Recurrent pilonidal sinus 

**Figure 3:**
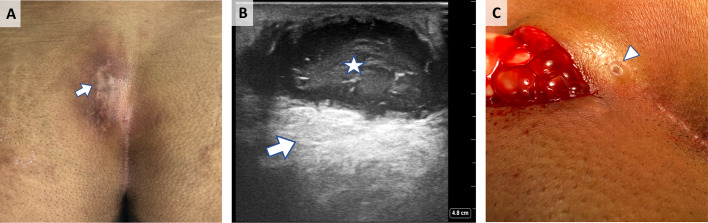
Recurrent pilonidal sinus with abscess. A)Site of previous operation, lateral to the natal cleft (arrow). B) Sonographic appearance of pilonidal abscess (star) with posterior acoustic enhancement (arrow). C)Incision and drainage. Central pit observed (arrowhead).

A 21-year-old male with a past history of pilonidal sinus surgery presented with a history of swelling at the site of the previous operation. POCUS was done by the attending proctologist and showed a recurrent pilonidal sinus with abscess formation. An incision and drainage were done along with the removal of the single pit (Figure 3). 

## Discussion

It is commonly believed that complementary investigations are rarely needed because the diagnosis of pilonidal sinus disease is a clinical one. However, sonography is an important diagnostic tool that is cost-effective, time-efficient, and able to visualize superficial structures proficiently. 

Ultrasonography is particularly useful in the evaluation of pilonidal sinus disease as it provides the ability to perform a soft tissue exam of the natal cleft and visualize the track in any plane necessary at the level above the coccyx 4. Ultrasonographic examination can provide gray-scale images of the affected site and color and pulse-waved Doppler 4. Using a high wave frequency transducer is ideal to examine areas near the skin surface. 

Ultrasonography can accurately evaluate the dimensions and shape of the pilonidal sinus pre-operatively and can therefore improve the management. Using ultrasonography, the surgeon can identify how far the tract is extending and the direction it is extending in, whether caudally or cranially. The surgeon can also identify whether the sinus is infected or has formed an abscess. With the information mentioned above provided by ultrasound imaging, the surgeon can tailor the surgical approach to each case and improve the overall outcome.

A study done Mentes O et al.^5^ showed the benefits of ultrasonography for detecting the borders of pilonidal sinus tissue in 73 male patients who were all examined and marked by the same surgeon. All patients also underwent scanning by the same radiologist, and power Doppler mode was used whenever needed. The ultrasonographic borders of the sinus tissue were similar to the borders marked by the surgeon in only 76.6% of patients (56 patients). The surgical approach was changed in 3 patients, and the incision line was changed in 14 patients after the information obtained by ultrasonography. In all of the 73 patients, surgically excised tissue and shape was fully compatible with the borders marked by ultrasonographic study and surgeons did not have the need to modify the surgical approach. Most fascinatingly, the recurrence rate was only 2.7% on 18-month follow-up visits 5. The study proved that pre-operative ultrasonography has a benefit over palpation and methylene blue staining when identifying the pilonidal sinus tract and its borders and branches 5. 

In brief, ultrasound in pilonidal sinus disease has a role in diagnosing, assessing the disease extent, presence or absence of abscess formation, and in detecting recurrent cases.

## Conclusion

Point of care ultrasound performed by surgeons is a useful non-invasive diagnostic tool in assessing pilonidal sinus. It can help tailor the surgical approach and map the extent of the disease preoperatively.

## Statement of Ethics

This publication complies with the guidelines for human studies and conducted ethically in accordance with the World Medical Association Declaration of Helsinki. Ethical approval was not required for this case report in accordance with the Dubai Scientific Research Ethics Committee policies. Witten Informed Consent for publication of the case report and any accompanied images was obtained from the patients.

## Conflict of Interest Statement

There are no conflict of interests to declare.

## Funding Sources

The authors did not receive any funding.

## Data Availability Statement

All data generated or analyzed during this case report are included in this article. Further enquiries can be directed to the corresponding author. 
